# Editorial Comment: Diagnostic ureteroscopy prior to nephroureterectomy for urothelial carcinoma is associated with a high risk of bladder recurrence despite technical precautions to avoid tumor spillage

**DOI:** 10.1590/S1677-5538.IBJU.2020.03.03

**Published:** 2020-02-20

**Authors:** M Baboudjian, K Al-Balushi, F Michel, F Lannes, A Akiki, S Gaillet, V Delaporte, E Ragni, H Toledano, G Karsenty, D Rossi, C Bastide, E Lechevallier, R Boissier, João Paulo Martins de Carvalho

**Affiliations:** 1 Aix-Marseille University La Conception University Hospital Department of Urology and Renal Transplantation Marseille France Department of Urology and Renal Transplantation, La Conception University Hospital, APHM, Aix-Marseille University, 13005, Marseille, France; 2 Aix-Marseille University Nord University Hospital, APHM Department of Urology Marseille France Department of Urology, Nord University Hospital, APHM, Aix-Marseille University, Marseille, France; 3 Aix-Marseille University La Conception University Hospital Department of Urology and Renal Transplantation Marseille France Department of Urology and Renal Transplantation, La Conception University Hospital, APHM, Aix-Marseille University, 13005, Marseille, France; 1 Hospital Federal Cardoso Fontes Serviço de Urologia Rio de JaneiroRJ Brasil Serviço de Urologia, Hospital Federal Cardoso Fontes, Rio de Janeiro, RJ, Brasil

## COMMENT

The use of flexible ureterorenoscopy for the diagnosis (DFU) of suspected lesions in the renal pelvis, calyx and other segments of the collecting system is common in urology. This elegant work tries to use a systematization of the surgical access in order to evaluate the possibility of tumor spillage in other urinary segments, when handling is diagnostic or therapeutic in urothelial tumors.

This is a single center study where patients underwent DFU prior to the procedure of radical nephro ureterectomy (RNU) from 2005 to 2017.

The great merit of this work was the definition of the following DFU operative technique:

1. A ureteropyelography with a ureteral catheter 7Fr with diluted contrast. Placement of a guide wire under visual and fluoroscopic control;
2. Placement of Ch 9-10 Peel-Away ureteral sheath on the guide wire. In case of UTUC in the ureter, the Peel- Away was placed just below the tumor and was then peeled to the urethral meatus;
3. A flexible ureteroscope was inserted in the ureteral sheath and selective cytology was performed close to the larger tumor;
4. A biopsy of the larger tumor was performed with a forceps biopsy
5. Complete exploration of the renal cavities was performed. Irrigation was provided with saline serum and a pressurized pump.
6. Retrograde ureteroscopy was performed and the ureteral sheath was removed with the ureteroscope. Drainage consisted of a mono-J + bladder catheter which was both removed on D1-D2 if no fever or significant bleeding was occurring.

Mostly important, no patient received postoperative endovesical instillation (EVI) after DFU.

RNU was performed after DFU, by open or laparoscopic access according to the surgeon's preference and bladder cuff excision without the endoscopic approach. Postoperative EVI began in March 2016.

All patients had preoperative endoscopic and tomographic staging to rule out metastases or multi-center tumors. Postoperative follow-up was performed according to the institution's protocol.

At the end, the patients were stratified into two groups before the RNU: those who underwent DFU (DFU+) and those who did not (DFU-). In a total of 171 patients who underwent RNU, 93 were included in the protocol: 70 - DFU+ and 23 - DFU -.

There was no statistical difference when comparing the baseline criteria between the two groups: age, tumor stage and tumor grade, postoperative pain analysis or positive surgical margins.

At the 35-month postoperative follow-up, DFU+ patients had a bladder recurrence (BR) up to 87%. No risk factor for BR other than DFU was found in a multivariate statistical analysis.

Despite the technical care, the odds ratio for DFU+ was 4.0. However, as reported by the authors, BR presented neoplasms of less aggressive behavior, low risk and superficial diseases, even if multifocal. There was no long-term impairment in overall survival, cancer-specific survival, and metastasis-free survival.

The reference cited by the authors places as risk factors for BR the presence of previous bladder neoplasia, high-grade neoplasms, urinary cytology prior to the RNU (1). Even so, this multivariate analysis did not use DFU as a risk factor for BR. Thus, the authors interpreted that factors linked to primary neoplasia could be more determinant than DFU itself for the presence of BR.

As reported, many studies cite DFU but without elucidating its technique, presence of ureteric sheaths, or confirmatory biopsy. Most importantly, in this study, all patients who had a history of previous resection of bladder neoplasms were excluded.

The authors also mention that despite RNU in high-grade urothelial tumors, intra-bladder recurrence was of low-grade neoplasms. This supports the need for further studies to decrease BR after DFU, such as intra-vesical chemotherapy, which is already performed after RNU.
